# Pathological features of COVID-19-associated liver injury—a preliminary proteomics report based on clinical samples

**DOI:** 10.1038/s41392-020-00406-1

**Published:** 2021-01-08

**Authors:** Ling Leng, Ruiyuan Cao, Jie Ma, Luye Lv, Wei Li, Yunping Zhu, Zhihong Wu, Manli Wang, Yiwu Zhou, Wu Zhong

**Affiliations:** 1 Stem Cell and Regenerative Medicine Lab, Translational Medicine Center, Department of Medical Science Research Center, Peking Union Medical College Hospital, Peking Union Medical College and Chinese Academy of Medical Sciences, Beijing, 100730 China; 2 National Engineering Research Center for the Emergency Drug, Beijing Institute of Pharmacology and Toxicology, Beijing, 100850 China; 3 State Key Laboratory of Proteomics, Beijing Proteome Research Center, National Center for Protein Sciences (Beijing), Beijing Institute of Life Omics, Beijing, 102206 China; 4 Institute of NBC Defense, Beijing, 102205 China; 5 State Key Laboratory of Virology, Wuhan Institute of Virology, Center for Biosafety Mega-Science, Chinese Academy of Sciences, Wuhan, 430071 China; 6 Department of Forensic Medicine, Tongji Medical College of Huazhong University of Science and Technology, Wuhan, 430030 China

**Keywords:** Infection, Infectious diseases, Systems biology

**Dear Editor**,

Almost one-half of COVID-19 patients have symptoms of liver injury.^1^ However, the molecular characteristics in the COVID-19 liver remain unknown. Therefore, the need for long-term medication for recovery make it imperative to study the infectivity and pathogenicity of SARS-CoV-2 in the liver. Here, we have identified significant proteomic changes for the first time in the liver of COVID-19 patients. We detected differential expression levels of several proteins that mediate characteristic cellular host responses elicited by SARS-CoV-2 infection in the liver.

Firstly, we co-stained the viral structural protein (spike) of SARS-CoV-2 and its functional receptor (ACE2), finding them enriched around the portal vein (PV) (Fig. 1a and Supplementary Fig. S1). Next, an integrated quantitative proteomics and phosphoproteomics approach was used to detect proteomic changes in the liver (Supplementary Fig. S2 and Supplementary Tables S1 and S2). A total of 1043 proteins (933 upregulated, 110 downregulated) and 200 phosphorylation sites (185 upregulated, 15 downregulated) were differentially expressed (BH-adjusted *p*-value < 0.01, Supplementary Fig. S3 and Supplementary Table S3) in the COVID-19 group as compared with the control group. Results showed that the levels of phase I drug metabolism enzymes (e.g. CPY3A4) and some phase III transporters were considerably downregulated (Supplementary Fig. S4a, b). Transporters of drug, heme, lipid, and nucleus were significantly increased, while bile acid and bile salt transporters were decreased (Supplementary Fig. S4c), indicating that the capacity of the substance transport system of the liver may be damaged in COVID-19 patients. Components of the urea cycle, phase II drug metabolism, liver-specific transcription factors, and functional markers were less affected (Supplementary Fig. S4a). The spatial specificity of liver function revealed that the homeostasis of glycolysis, lipogenesis, and heme and ketone biosynthesis in the central vein (CV), and gluconeogenesis, fatty acid oxidation, and cholesterol biosynthesis in the PV of liver was imbalanced in COVID-19 (Supplementary Fig. S5a, b). LEPR-JAK1-STAT3 and ADIPOR-AMPK were activated, which mediate β-oxidation activation, thereby leading to insulin resistance (Fig. 1b and Supplementary Fig. S6a). Moreover, the insulin signaling was activated, which activated both the mTORC1 (AKT2-P, mTOR-P, and EIF4E2) and mTORC2 (mTOR-P, PRKC, ACACA-P, and ACACB-P) pathways that lead to protein synthesis, cytoskeletal organization, and fatty acid biosynthesis (Supplementary Fig. S6a). Remarkably, these differentially expressed proteins were associated with diabetes mellitus and plasma HDL cholesterol (HDL-C) disease, suggesting the risk for insulin resistance and fatty liver in COVID-19 patients (Supplementary Fig. S5a). Further analysis suggested that the fatty acid transporters (CD36, SLC27A1, and SLC27A4), fatty acid acetylase (CPT1), and lipid synthesis-associated enzymes (AGPAT1 and AGPAT5) were highly expressed in the liver of patients with COVID-19, possibly leading to the generation of lipid droplets through the activation of the β-oxidation pathway (Fig. 1c). Enrichment of fat droplets in the CV of COVID-19 livers was observed (Fig. 1d and Supplementary Fig. S7). In addition, low expression of the LDL transporter (APOB) in livers from COVID-19 patients may also cause the accumulation of lipid in the liver (Fig. 1d). Further, proteins associated with epithelial-mesenchymal transition (EMT) were upregulated, while some proteins associated with mesenchymal-epithelial transition (MET) were downregulated in the COVID-19 group (Supplementary Fig. S5c), indicating that changes in the metabolic pathways may lead to liver fibrosis of the COVID-19 patients.

**Fig. 1:**
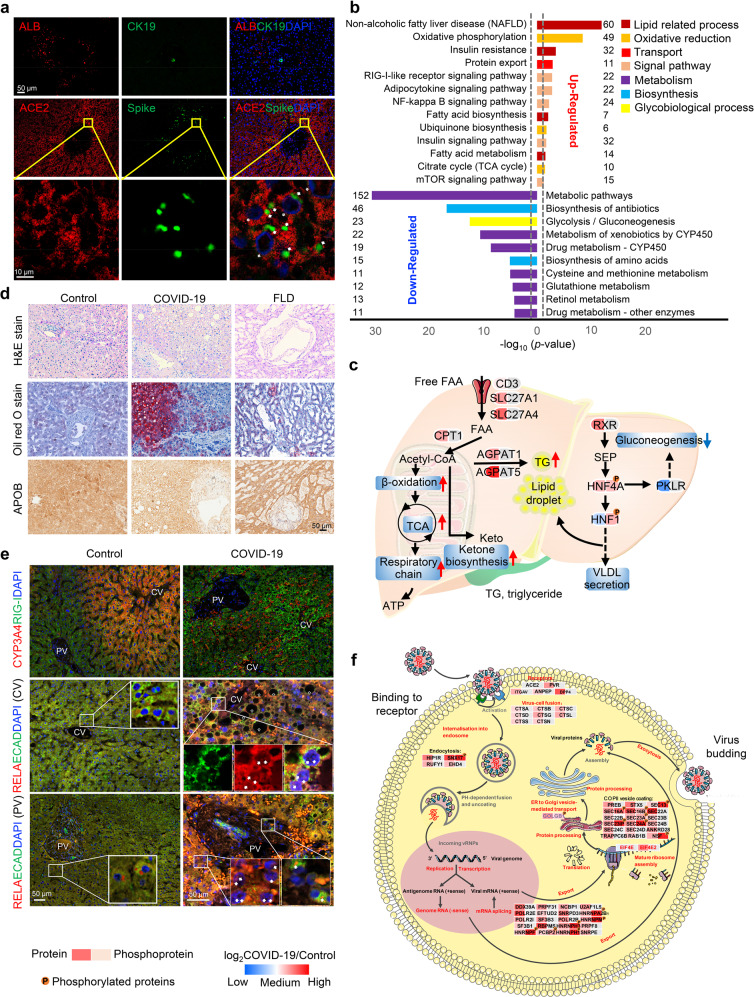
Functional characterization of liver from patients with COVID-19. **a** Immunofluorescence analyses of ALB, CK19, ACE2, and spike proteins expressed in liver tissues from COVID-19 patients and control individuals (scale bar: 50 and 10 μm). Solid and hollow arrows point to the virus proteins (spike) and damaged nucleus, respectively. Cytokeratin-19 (CK19) is the bile duct marker at PV. **b** KEGG pathway enrichment analysis of differentially expressed proteins in the liver tissue of COVID-19 patients vs. the control group. The color bars represent the functional categories. **c** Scheme showing dysregulation of metabolism processes and mechanisms of hepatocellular lipid droplet accumulation. Alterations are defined by up- or downregulated proteins or phosphorylation sites with red and blue boxes according to log_2_ (ratio of average protein abundance in COVID-19 vs. the control group). **d** Hematoxylin & eosin staining, oil red O staining, and immunohistochemistry of APOB in liver tissues from patients diagnosed with COVID-19, fatty liver disease (FLD), and control individuals (scale bar: 50 μm). **e** Immunofluorescence analyses of CYP3A4, RIG-I, RELA, and ECAD proteins expressed in liver tissues from COVID-19 patients and control individuals (scale bar: 50 μm). Arrowheads point to RELA proteins that enter the nucleus. **f** Schematic representation of the process of SARS-CoV-2 infection, including virus entry into the host cell, endocytosis, virus genome replication and transcription, mRNA splicing and translation, protein processing and assembly, and exocytosis and release from the host cell

Several inflammatory factors were detected differentially expressed in the lung tissues of COVID-19 patients, suggesting the presence of a cytokine storm in the lung.^2^ Our results showed that the highly expressed proteins in the COVID-19 livers were enriched in RIG-I, TNF, and IL1R signals, which finally integrated into the NF-κB-mediated inflammatory pathway (Fig. 1b and Fig. S6a). Further, we verified RIG-I and IPS-1 with high expression and observed RELA entry into the nucleus in hepatocytes around both CV and PV (Fig. 1e). Notably, the expression of interferon (IFNα) and interleukin (IL8) downstream of the NF-κB/RELA pathway was significantly higher in the COVID-19 hepatocytes than in the control group (Supplementary Fig. S6b). Moreover, the noncanonical NF-κB/RELB pathway was found increased in the livers of COVID-19 patients (Supplementary Fig. S6a).

Next, we explored the molecular events of viral pathology in the liver, and found the COVID-19 samples were enriched in proteins involved in transcription, translation, transport, and viral processes (Supplementary Fig. S8a). Analysis of viral processes suggested a dynamic process of SARS-CoV-2 infection in the liver (Supplementary Fig. S9a and Fig. 1f). For example, we verified human coronavirus 229E/HCoV-229E receptor (ANPEP) and Middle East respiratory syndrome coronavirus receptor (DPP4) highly expressed in the COVID-19 liver tissues (Supplementary Fig. S9b). DPP4 was speculated to be the potential receptor for SARS-CoV-2.^3^ The upregulation of virus-associated proteins may also cause changes in liver function, observed by an integrated interactome network between the virus-associated proteins in Supplementary Fig. S9a and differentially expressed functional proteins of the liver (Supplementary Fig. S9c). For example, lipid, energy, and drug metabolism were closely related to the processes of response to the virus, including viral genome replication, viral transcription, and viral entry (Supplementary Fig. S9c). The dynamic change of EMT may be regulated by the process of viral entry into the host and viral genome replication (Supplementary Fig. S9c).

A previous report has identified SARS-CoV-2-host interacting proteins (332 proteins) as potential drug targets.^4^ Of these, 275 were identified in the COVID-19 liver samples, including 70 (with 30 phosphorylated) significantly upregulated and two (with one phosphorylated) significantly downregulated proteins (Supplementary Fig. S8b). A protein–protein interaction network (Supplementary Fig. S9c) was constructed within the 275 proteins, along with the viral proteins, virus process-related proteins (Supplementary Fig. S9a), and liver function-related proteins (Supplementary Fig. S4-5). The top 22 strong-interacting proteins included nine transport-related proteins, one tRNA synthetase, one tRNA-modifying enzyme that is involved in viral translation, two membrane organization-related proteins, six proteins involved in protein assembly, one protein involved in protein localization, and one polio virus receptor (PVR) (Supplementary Fig. S9c and Supplementary Table S4). PVR, which was highly expressed in COVID-19 liver tissues, showed strong interaction with the open reading frame protein (ORF8) of SARS-CoV-2 (Fig. 1f and Supplementary Fig. S9c), indicating that virus-interacting proteins identified in SARS-CoV-2-infected livers may be potential targets for medical intervention and drug research. In addition, 10 medium and 169 weak interactions between viral proteins and the proteins in the COVID-19 livers were found in the interaction network (Supplementary Table S4).

Further, a comprehensive scoring table was constructed for the proteins involved in virus-related biological process, based on the protein expression profile, fold change ratio (COVID-19 to control, both protein and phosphorylation sites), the *p*-value score (COVID-19 to control), the interaction information with SARS-CoV-2 proteins, and liver-specific and transport function proteins (Supplementary Fig. S10 and Supplementary Table S5). The proteins in the scoring table were listed as potential drug targets. These proteins scored “A”, indicating their relatively superior potential as drug targets for the treatment of COVID-19-mediated liver diseases.

In conclusion, we suggest that liver injury in COVID-19 may be directly associated with virus infection. Further, patients with COVID-19 may be prone to suffer from FLD. Patients with fatty liver should be paid more attention to protect the liver from further steatosis. In addition, risk of hepatitis should be noticed due to activation of the immune pathway. Finally, 202 potential therapeutic drug targets are presented to develop possible countermeasures against COVID-19 liver damage.

## Supplementary information

Supplementary Materials

Supplementary Table S2

Supplementary Table S3

Supplementary Table S4

Supplementary Table S5

Supplementary Table S6

## Data Availability

All proteomics raw data have been deposited to the ProteomeXchange Consortium via the iProX partner repository with the dataset identifier PXD019968 (http://proteomecentral.proteomexchange.org/cgi/GetDataset?ID=PXD019968).
