# Supplementary material for: Pathological features of COVID-19-associated liver injury—a preliminary proteomics report based on clinical samples
Source: Signal Transduct Target Ther. 2021 Jan 8;6:9. doi: 10.1038/s41392-020-00406-1 (PMC7791959; doi:10.1038/s41392-020-00406-1)
Supplement: Supplementary file 1 — Supplementary Materials [file 41392_2020_406_MOESM1_ESM.pdf]

Supplementary Materials for

Pathological Features of COVID-19 Associated Liver Injury - A Preliminary Proteomics Report Based on Clinical Samples

Ling Leng[†], Ruiyuan Cao[†], Jie Ma[†], Luye Lv[†], Wei Li[†], Yunping Zhu[*], Zhihong Wu[*], Manli Wang[*], Yiwu Zhou[*] and Wu Zhong[*]

Correspondence to: Yunping Zhu (zhuyunping@gmail.com), Zhihong Wu (orthoscience@126.com), Manli Wang (wangml@wh.iov.cn), Yiwu Zhou (zhouyiwu@outlook.com) or Wu Zhong (zhongwu@bmi.ac.cn).

This PDF file includes:

Supplementary Materials and Methods

Supplementary Figures S1 to S10

Supplementary Table S1

Other Supplementary Materials for this manuscript include the following:

Supplementary Table S2 to S6

Supplementary Materials and Methods

Human Subjects

Three liver samples were obtained after autopsy from three clinically confirmed patients with COVID-19 pneumonia within four hours after death. The samples were obtained from the upper left lateral lobes. The decrease of albumin (ALB) made by liver suggested the dysregulation of liver function (Supplementary Table S1). Pathological sections of control human livers without COVID-19 pneumonia (fatty liver, female, 61; normal liver, female, 70) were used for validation. All samples were obtained from Wuhan Jinyintan Hospital, Wuhan, China. This study was approved by the Medical Ethics Committee of Wuhan Jinyintan Hospital (NO. AF/SC-13/01.0), and written informed consent was obtained from the patients' families. Further details can be found in Supplementary Table S1. Laboratory confirmation of SARS-CoV-2 infection was performed at Jinyintan and Wuhan Central Hospital and Wuhan Institute of Virology, Wuhan, China. SARS-CoV-2 viral RNA was confirmed by real-time quantitative RT-PCR.

The datasets produced by Jiang *et al.*[1] were used here as the proteome and phosphoproteome profile for control liver tissue. To ensure the consistency between samples, the non-tumor liver tissues from older individuals were selected (aged from 70~80). The MS/MS raw files generated from six non-tumor liver tissues (Sample No.: L032, L038, L044, L058, L064, and L103, details refer to the Supplementary Table 1 from the work of Jiang *et al.*[1]), were downloaded from the iProX repository (https://www.iprox.org/page/project.html?id=IPX0000937000), and reprocessed using the same data analysis workflow as that for COVID-19 samples.

Hematoxylin/Eosin staining, histology, and immunofluorescence

Liver tissue samples from patients with or without COVID-19 pneumonia were washed

twice with cold 1 × PBS and fixed in 10% neutral buffer formalin solution for 48 h at 4 °C. After rinsing with cold 1 × PBS, the liver samples were embedded in paraffin following standard protocols and sectioned at a thickness of 4 μm using a microtome. After deparaffinization and rehydration, antigen retrieval was performed with tris-EDTA buffer (10 mM Tris base, 1 mM EDTA solution, and 0.05% Tween 20 (pH 9.0). The sections were washed three times with water and stained with hematoxylin and eosin (H&E).

For immunohistochemical staining, the sections were deparaffinized, and boiled in a microwave for 12 min with the antigen retrieval buffer. Thereafter, the samples were cooled and endogenous catalase was removed using 0.3 % H_2O_2 for 30 min. The sections were blocked using normal horse serum in Tris-buffered saline for 1 h and with the Avidin/Biotin Blocking Kit and stained with antibodies overnight at 4 °C. After incubation with a secondary antibody, the sections were stained.

For immunofluorescence staining, cells were fixed in 4% formaldehyde for 20 min and washed in PBS. The cells were treated with 0.25% Triton X-100 for 20 min, blocked in 10% serum for 1 h at 25 °C, and incubated with primary antibodies overnight at 4 °C. After being incubated for 1 h at 25 °C with secondary antibodies and counterstained with DAPI, the sections were sealed with Fluoro-Gel for photography. The negative controls were incubated with secondary antibody alone. Microscopy images were photographed at 4×, 20×, and 40× magnification and analyzed using InForm 2.2.

Oil red O staining

After the liver tissue samples were fixed in 4% paraformaldehyde for 48 h and treated with 30% sucrose, 4 μm cryosections were prepared. The slides were washed with 70% isopropanol for 5 min. Thereafter, the samples were stained with oil red O for 30 min at 25 °C. After washing the slides three times with dH_2O and twice with 70% isopropanol,

the images of stained liver samples were taken using Vectra. Images were captured at 4 ×, 20 ×, and 40 × magnification and analyzed using InForm 2.2.

Mass spectrometry

100 mg liver tissue samples from patients with COVID-19 were freezingly homogenized in a BSL-III laboratory. After centrifugation at 14,000× *g* for 10 min at 4 °C, the supernatants were reduced by adding tributylphosphine (final concentration, 5 mM), followed by vortexing for 10 min at 25 °C. After centrifugation at 14,000× *g* for 30 min at 25 °C, the supernatants were transferred to a clean tube, and 100 μg samples were added to a 10 kDa ultrafiltration tube with 400 μL of urea buffer (8 M urea, 150 mM Tris HCl, pH 8.0). After centrifugation at 12,000 × *g* for 10 min at 25 °C, the liquid in the collection tube was discarded, and the aforementioned steps were repeated three times. For peptide extraction, 25 μg samples (from the supernatants) were solubilized in 10 mM DTT at 37 °C for 4 h. Samples were added with 50 mM IAA and kept in the dark for 30 min. All samples were collected, and the suspension was removed after centrifugation for 12,000× *g*. Thereafter, 100 μL UA was added and the suspension was removed after centrifugation twice. NH_4HCO_3 (50 mM) was added, and the suspension was removed after centrifugation for three times. The final digestion was done using trypsin at 37 °C overnight, at a ratio of 1:50 = enzyme:substrate.

The peptide samples were placed in buffer A (98% ddH2O, 2.0% acetonitrile, ammonia water, pH = 10). After separation using an XBridge® peptide BEH C18 high performance liquid chromatographic column (130 Å, 3.5 μm, 4.6 mm × 150 mm), elution buffer B (98% acetonitrile, 2.0% ddH2O, pH = 10) was used to separate for 46 min (flow rate 1 mL/min). A tube of components was collected every 1 min, and a total of 40 separated components were collected. The samples were combined into 6 tube components, placed in a rotary vacuum dryer, and freeze dried at −20 °C for standby.

The peptide mixtures were analyzed using an Orbitrap Fusion Lumos Mass Spectrometer equipped with an Easy-nLC nanoflow liquid chromatography system. The peptide mixture resolved in buffer A (0.1% formic acid (FA)) were loaded onto a 2-cm self-packed trap column (150-μm inner diameter, ReproSil-Pur C18-AQ, 1.9 μm) using buffer A and separated on a 150-μm-inner-diameter column with a length of 15 cm (ReproSil-Pur C18-AQ, 1.9 μm) over a 90-min gradient (buffer A, 0.1% FA in water; buffer B, 0.1% FA in ACN) at a flow rate of 600 nL/min (0–11 min, 8–15% B; 11–61 min, 15–28% B; 61–83 min, 28–45% B; 83–84 min, 45–95% B; and 84–90 min, 95% B).The Orbitrap Fusion Lumos was set to the OT–IT mode. For a full mass spectrometry survey scan, the target value was 5e5 and the scan ranged from 300 to 1,400m/z at a resolution of 120,000 and a maximum injection time of 50 ms. For the MS2 scan, a duty cycle of 3 s was set with the top-speed mode. Only spectra with a charge state of 2–6 were selected for fragmentation by higher-energy collision dissociation with normalized collision energy of 32%. The MS2 spectra were acquired in the ion trap in normal mode a maximum injection time of 12 s.

Phosphor-peptide enrichment and fractionation

The procedure of phosphor-peptide segment was performed according to the manufacturer's instructions. Briefly, after preparing the column, the equilibrated TiO_2 Spin Tip and adaptor were transferred into a new 2 mL microcentrifuge tube. A total of 150 μL of suspended peptide sample was added to the spin tip and centrifuged at 1,000× *g* for 5 min. The sample was reapplied in the microcentrifuge tube to the Spin Tip. After centrifugation at 1,000× *g* for 5 min, the steps were repeated once. Next, the TiO_2 Spin Tip and adaptor were transferred into the collection tube. After washing the column with 20 μL of Binding/Equilibration Buffer, the microcentrifuge tube was centrifuged at 3,000× *g* for 2 min. To wash the column, 20 μL of Wash Buffer was added, and the

microcentrifuge tube was centrifuged at 3,000× *g* for 2 min. The Wash Column steps were repeated once. Thereafter, the column was washed with 20 μL of LC-MS grade water and centrifuged at 3,000× *g* for 2 min. Next, 50 μL of Phosphopeptide Elution Buffer was added and the samples were centrifuged at 1,000 × *g* for 5 min; the step was repeated once. The eluate was dried in a vacuum concentrator to remove the Phosphopeptide Elution Buffer and suspended in 10 μL of 0.1% formic acid for MS analysis. The phosphopeptide mixtures were analyzed using an Orbitrap Fusion Lumos Mass Spectrometer equipped with an Easy-nLC nanoflow liquid chromatography system.

Proteomics MS/MS data processing and bioinformatics analysis

All raw files from mass spectrometry analysis for both control and SARS-CoV-infected liver tissues were analyzed using the MaxQuant software (version 1.6.5.0).[2] Proteins were identified by searching against a database containing the SwissProt human sequences (accessed on April 17, 2020, containing 20,367 human proteins) with the common contaminants included in MaxQuant. Peptides were identified using a precursor mass tolerance of 4.5 ppm and a fragment mass tolerance of 20 ppm. Cysteine carbamidomethylation was set as the fixed modification, and N-terminal acetylation and methionine oxidation served as variable modifications. For the phospho-proteome data, serine (S), threonine (Y), and tyrosine (T) phosphorylation were searched as variable modifications. Two and three missed cleavages were allowed for proteome and phospho-proteome data, respectively, and trypsin was set as the reference enzyme. Automatic target and reverse database searches were enabled with a maximum false discovery rate of 0.01 for peptide and protein identification. A minimum Andromeda score of 40 was required for modified peptides and a site localization probability of 0.75 was used as threshold for phosphosite localization. Protein quantification was performed

according to the intensity-based absolute quantification method iBAQ[3] as implemented in MaxQuant, and the median normalization was used to reduce the biases between experiments.

For protein expression in liver tissue from patients with COVID-19 and control samples, an unpaired, moderated *t*-test, as implemented in the limma Package in R software (V3.38.3), was performed for analysis of differences in expression. The Benjamini-Hochberg (BH) procedure was also implemented, and *p*-values lower than 0.05 were considered statistically significant ($*p < 0.05$, $**p < 0.01$). The significance of differentially expressed proteins or phosphorylation sites were defined with BH adjusted p-value < 0.01 and a fold change of COVID-19/Control > 2 (significantly upregulated) or < 1/2 (significantly downregulated), and the proteins or sites should be identified in at least 50% of the COVID-19 and control samples. Then, the differentially expressed proteins and phosphorylation sites are presented using volcano plot analysis.

Protein identifications were annotated with the online tool DAVID (https://david.ncifcrf.gov/)[4] according to biological processes, cellular components, and molecular functions within the GO[5] and KEGG[6] pathway analyses. The protein-protein interactome network was built using Cytoscape (version 3.7.2)[7] and the protein-protein associations were retrieved from the STRING database.[8]

DATA AVAILABILITY

All proteomics raw data have been deposited to the ProteomeXchange Consortium via the iProX[9] partner repository with the dataset identifier PXD019968.

References

1. Jiang, Y. *et al.* Proteomics identifies new therapeutic targets of early-stage hepatocellular carcinoma. *Nature* **567**, 257-261 (2019).
2. Cox, J. & Mann, M. MaxQuant enables high peptide identification rates, individualized p.p.b.-range mass accuracies and proteome-wide protein quantification. *Nat Biotechnol* **26**, 1367-1372 (2008).
3. Schwanhausser, B. *et al.* Global quantification of mammalian gene expression control. *Nature* **473**, 337-342 (2011).
4. Huang da, W., Sherman, B. T. & Lempicki, R. A. Bioinformatics enrichment tools: paths toward the comprehensive functional analysis of large gene lists. *Nucleic Acids Research* **37**, 1-13 (2009).
5. Ashburner, M. *et al.* Gene ontology: tool for the unification of biology. The Gene Ontology Consortium. *Nat Genet* **25**, 25-29 (2000).
6. Ogata, H. *et al.* KEGG: Kyoto Encyclopedia of Genes and Genomes. *Nucleic Acids Research* **27**, 29-34 (1999).
7. Shannon, P. *et al.* Cytoscape: a software environment for integrated models of biomolecular interaction networks. *Genome Res* **13**, 2498-2504 (2003).
8. Szklarczyk, D. *et al.* The STRING database in 2017: quality-controlled protein-protein association networks, made broadly accessible. *Nucleic Acids Res* **45**, D362-D368 (2017).
9. Ma, J. *et al.* iProX: an integrated proteome resource. *Nucleic Acids Res* **47**, D1211-D1217 (2019).

Supplementary figures

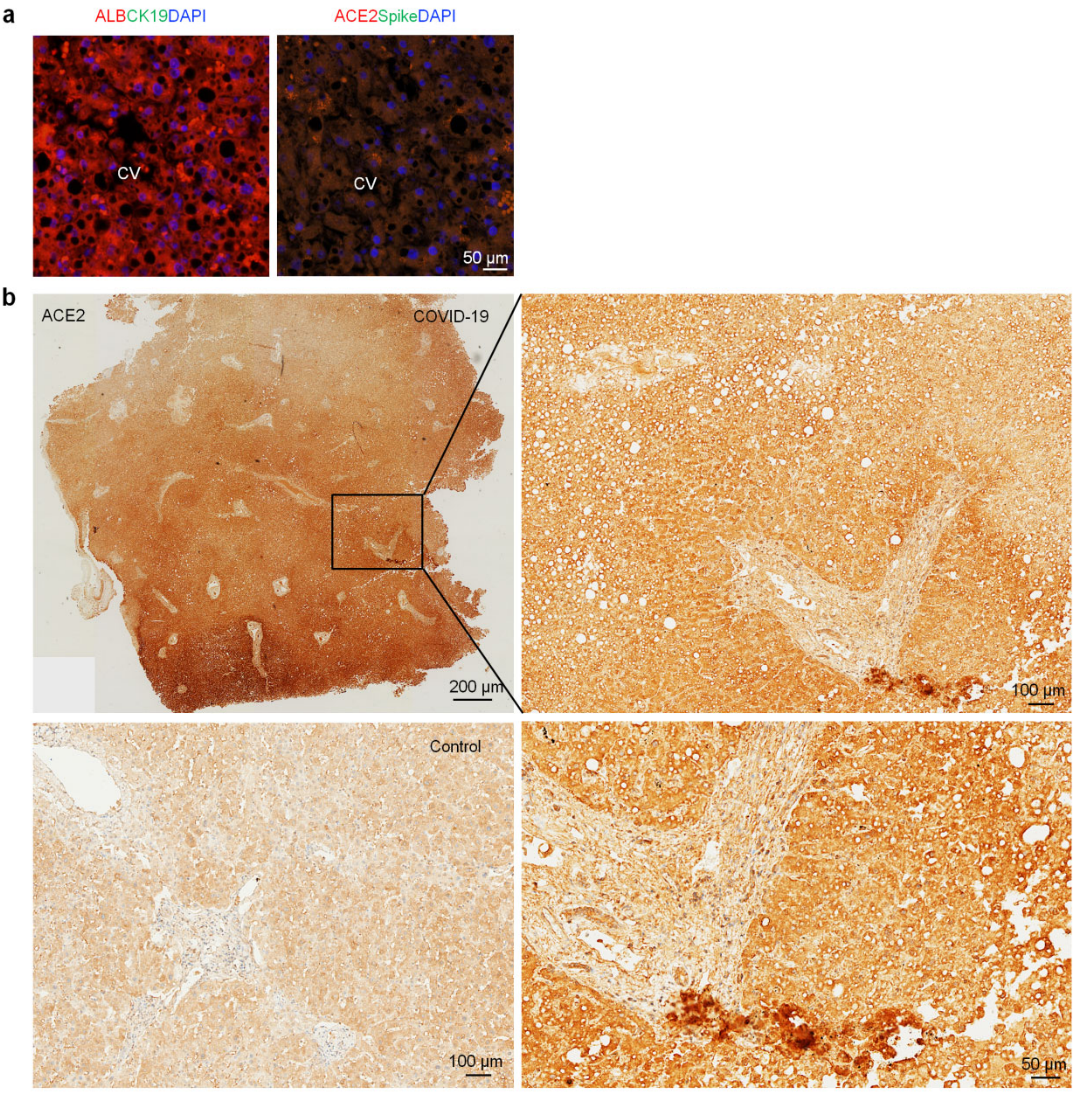

Fig. S1 Verification of human liver tissue affected by SARS-CoV-2. **a** Immunofluorescence analyses of ALB, CK19, ACE2, and spike proteins expressed in the liver tissues of patients diagnosed with COVID-19 and control individuals. (scale bar: 50 μm). **b** Immunohistochemistry of ACE2 in the liver tissues of patients diagnosed with COVID-19 and control individuals (scale bar: 50, 100 and 200 μm).

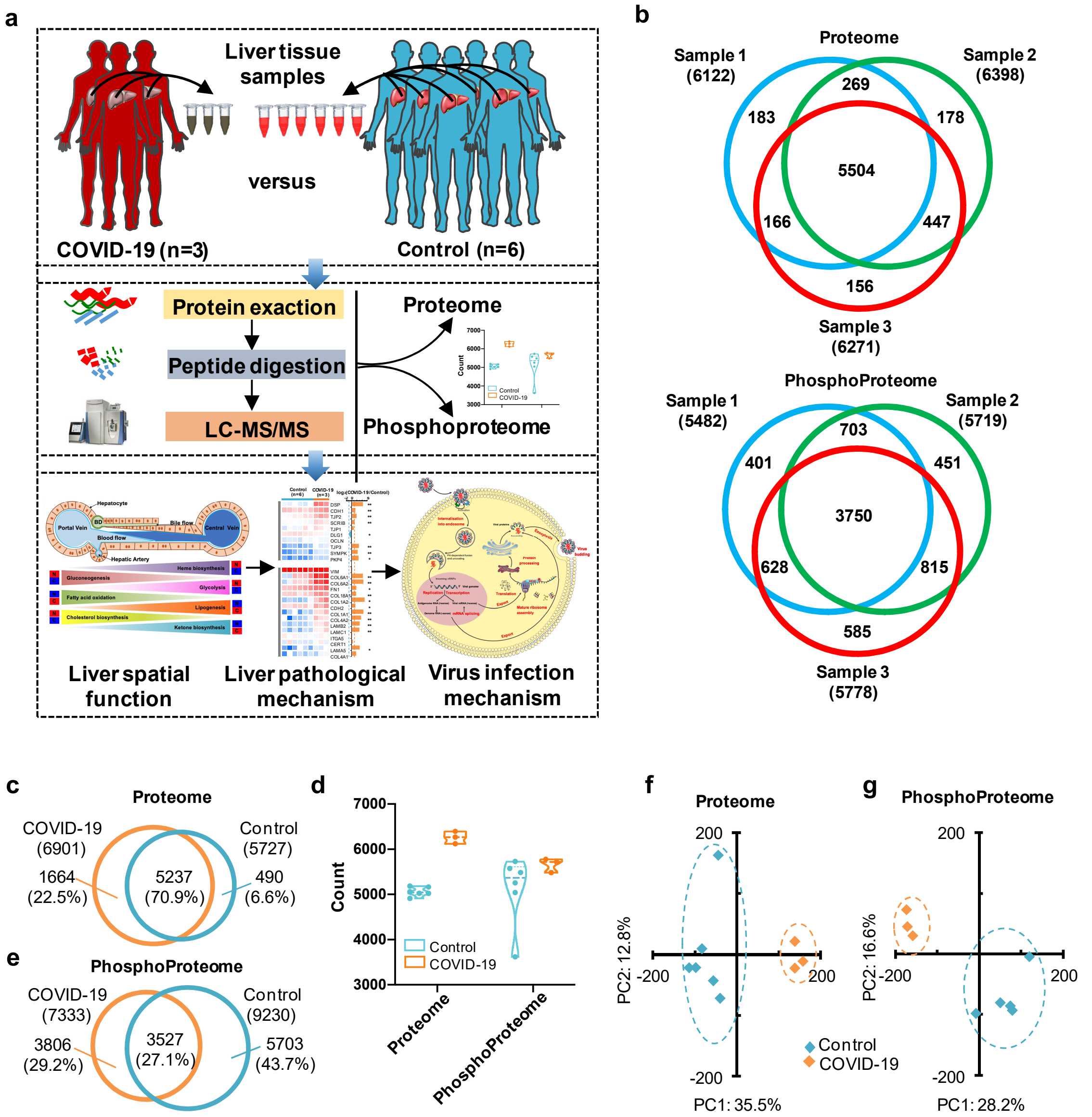

Fig. S2 Quantitative proteomic and phosphoproteome profile of SARS-CoV-2 infected human liver tissue. **a** Schematic of proteomics analysis used to evaluate liver tissue from patients diagnosed with COVID-19. **b** Venn diagrams showed the reproducibility of proteins and phosphor-sites in liver tissues of patients diagnosed with COVID-19 and control individuals. Overlaps of the proteome (**c**) and phosphoproteome (**e**) identified in liver tissues from patients diagnosed with COVID-19 and control individuals. **d** Count of protein and phosphorylation sites in liver tissues from patients diagnosed with COVID-19 and control individuals, respectively. Principal component analysis of COVID-19 and control samples based on the quantitative proteome (**f**) and

phosphoproteome (**g**) profiles of liver tissues from patients diagnosed with COVID-19 and control individuals.

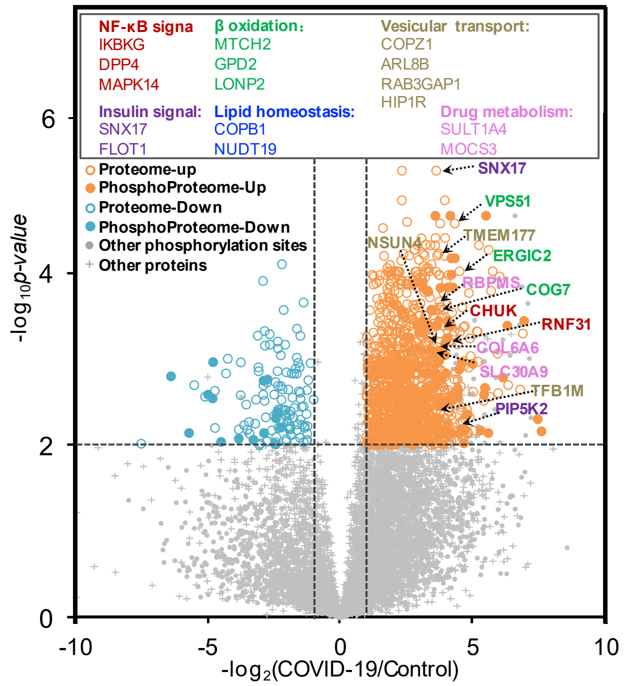

Fig. S3 Volcano plots of $-\log_{10}$ *p*-value vs. $\log_2$ ratio of protein and phosphorylation site abundance comparisons between livers from control individuals and those diagnosed with COVID-19, respectively. Proteins outside the significance threshold lines ($-\log_{10}$ (p-value) > 2 and $\log_2$ (COVID-19/Control) > 1 or < -1) are in orange (upregulated) or cerulean (downregulated). Hollow and solid circles represent the proteins of proteome profile and phosphorylation sites, respectively.

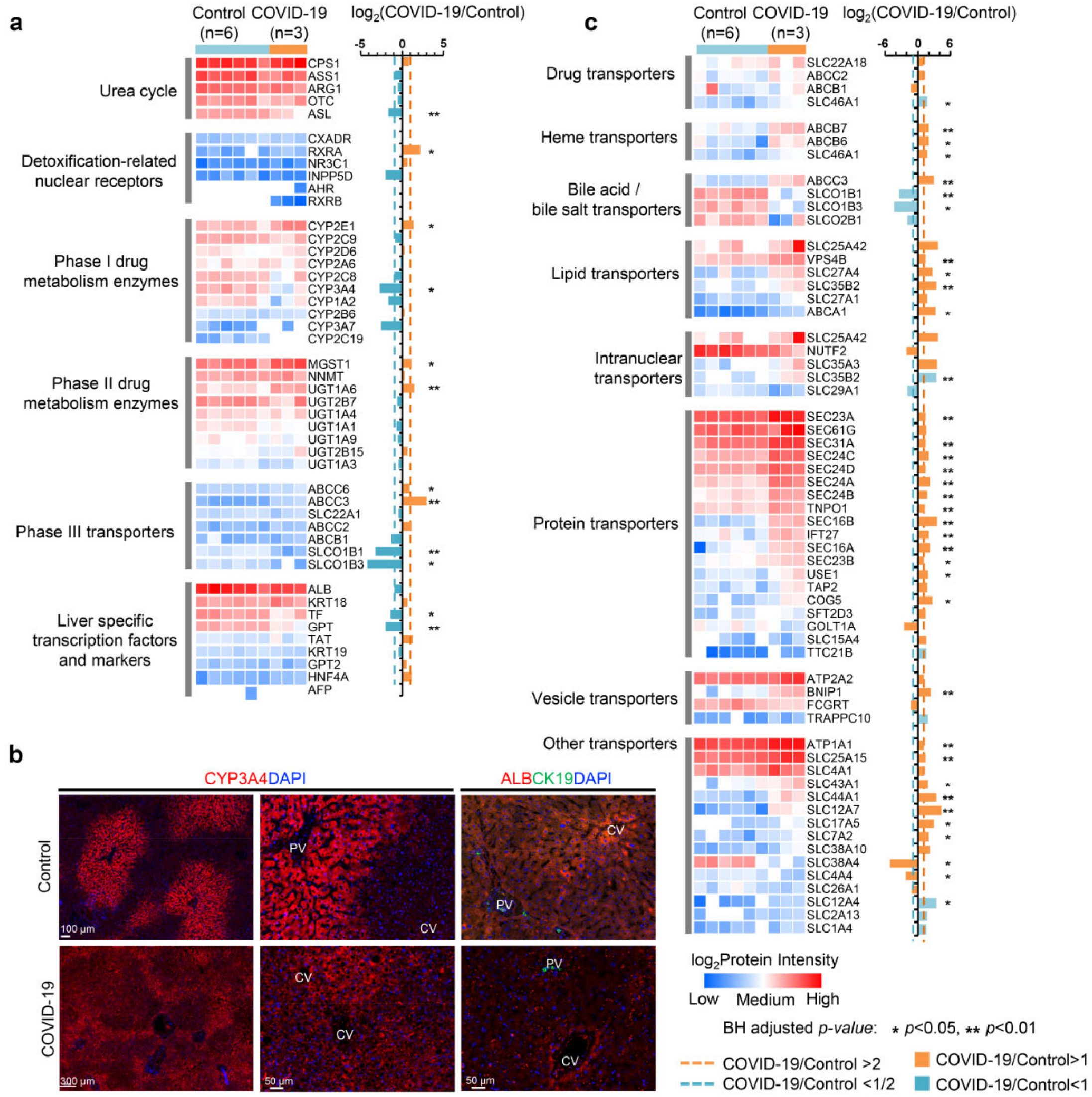

Fig. S4 Functional characterization of the liver of patients diagnosed with COVID-19. Liver function (**a**) or transporter (**c**) analysis of differentially expressed proteins between COVID-19 and control samples. Columns on the left of the heatmap represent different functional categories. The right of the heatmap presents the gene names. Red and blue boxes indicate the $\log_2$ of the intensities of the enriched or depleted proteins, respectively. Histogram analysis of the ratio of the protein intensities from COVID-19 compared to the control groups. Y-axis represents the $\log_2$ COVID-19/Control. The dotted lines represent the threshold of $\log_2$ COVID-19/Control. **b** Immunohistochemistry of CYP3A4, ALB, and CK19 in the liver tissues of patients diagnosed with COVID-19 and control individuals (scale bar: 50, 100, and 300 μm).

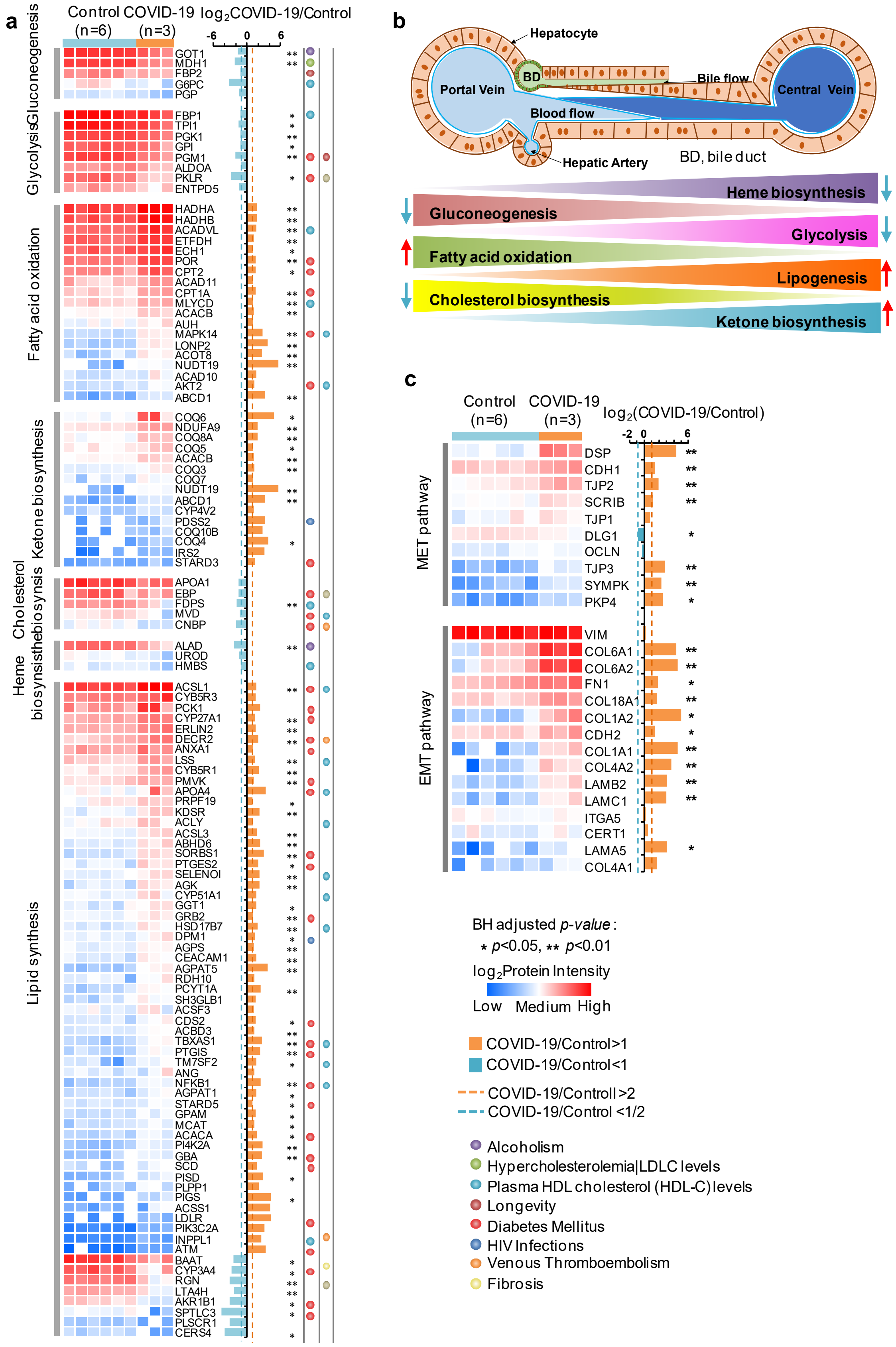

Fig. S5 Metabolic characterization of the liver of patients diagnosed with COVID-19. **a** Metabolic function analysis of differentially expressed proteins between COVID-19 and

control samples. Columns on the left of the heatmap represent different functional categories. The right side of the heatmap shows the gene names. Red and blue boxes indicate the log_2 of the intensities of enriched or depleted proteins, respectively. Histogram analysis of the ratio of protein intensities from COVID-19 compared to the control groups. Y-axis represents log_2 COVID-19/Control. The dotted lines represent the threshold of log_2 COVID-19/Control. Cycles with colors represent different kinds of disease. **b** Schematic of liver function arranged across the lobule. The changes in various functions on distinct metabolic zones in liver tissues from patients diagnosed with COVID-19. Triangles with different colors represent different functions. The colors from light to deep represent the enrichment degree of corresponding functions (from low to high) in the CV or PV. The red and blue arrows represent the metabolic processes highly and lowly enriched in liver tissues with COVID-19, respectively. **c** MET/EMT analysis of differentially expressed proteins between COVID-19 and control. Columns on the left of the heatmap represent different function categories. The right of the heatmap presents the gene names. Red and blue boxes indicate the log_2 of the intensities of the enriched or depleted proteins, respectively. Histogram analysis of the ratio of protein intensities from COVID-19 compared to the control groups. Y-axis represents the log_2 COVID-19/Control. The dotted lines represent the threshold of log_2 COVID-19/Control.

a

b

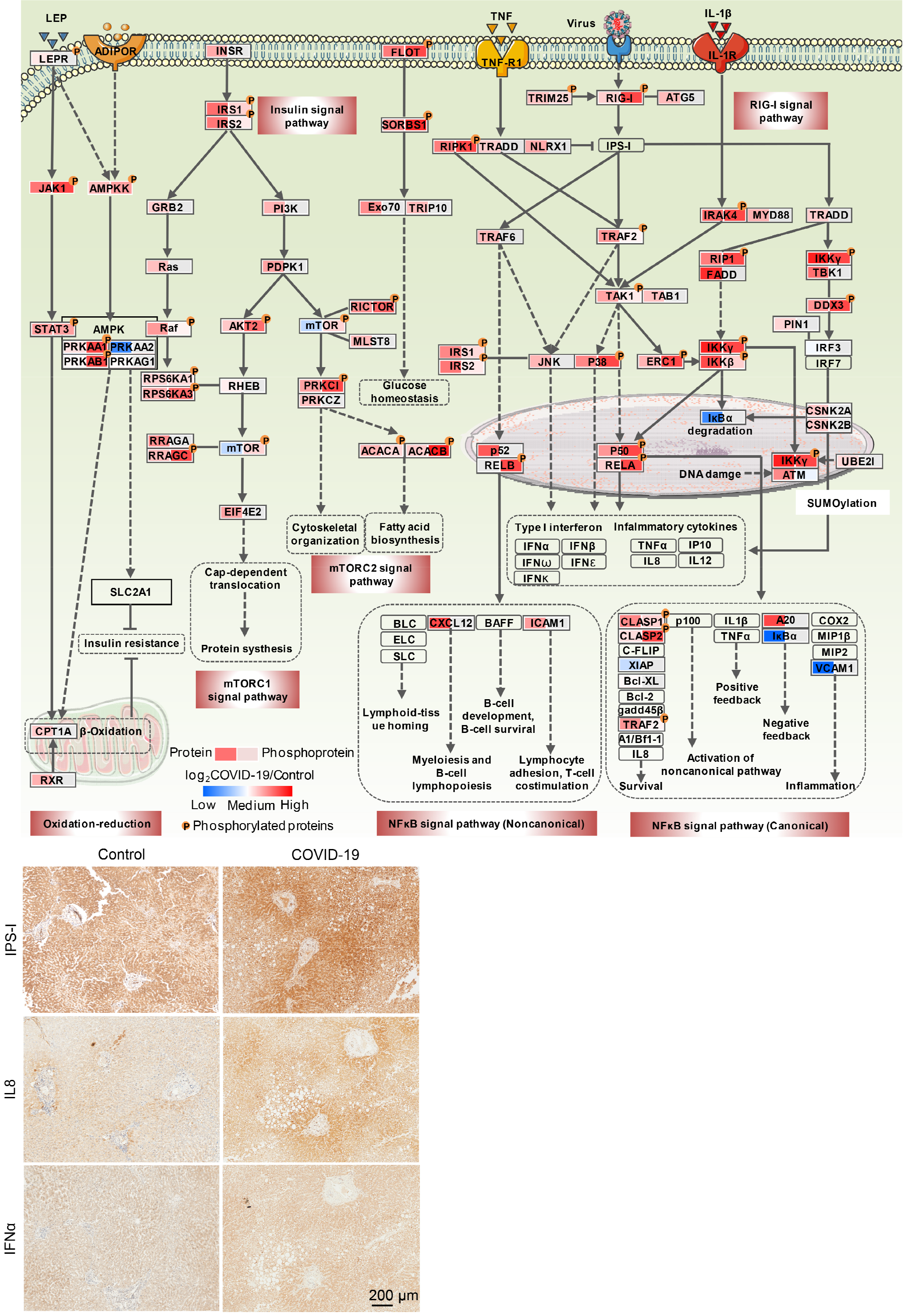

Fig. S6 Signaling pathways involved in metabolism and inflammation in the liver tissue of patients with COVID-19. **a** Overview of biological signaling pathways based on integrated proteome analysis. Alterations are defined by up- or downregulated proteins and phosphorylation sites with red and blue boxes according to $\log_2$ COVID-19/Control.

b Immunohistochemistry of IPS-1, IL8 and IFNα in liver tissues from patients with COVID-19 and control individuals (scale bar: 200 μm).

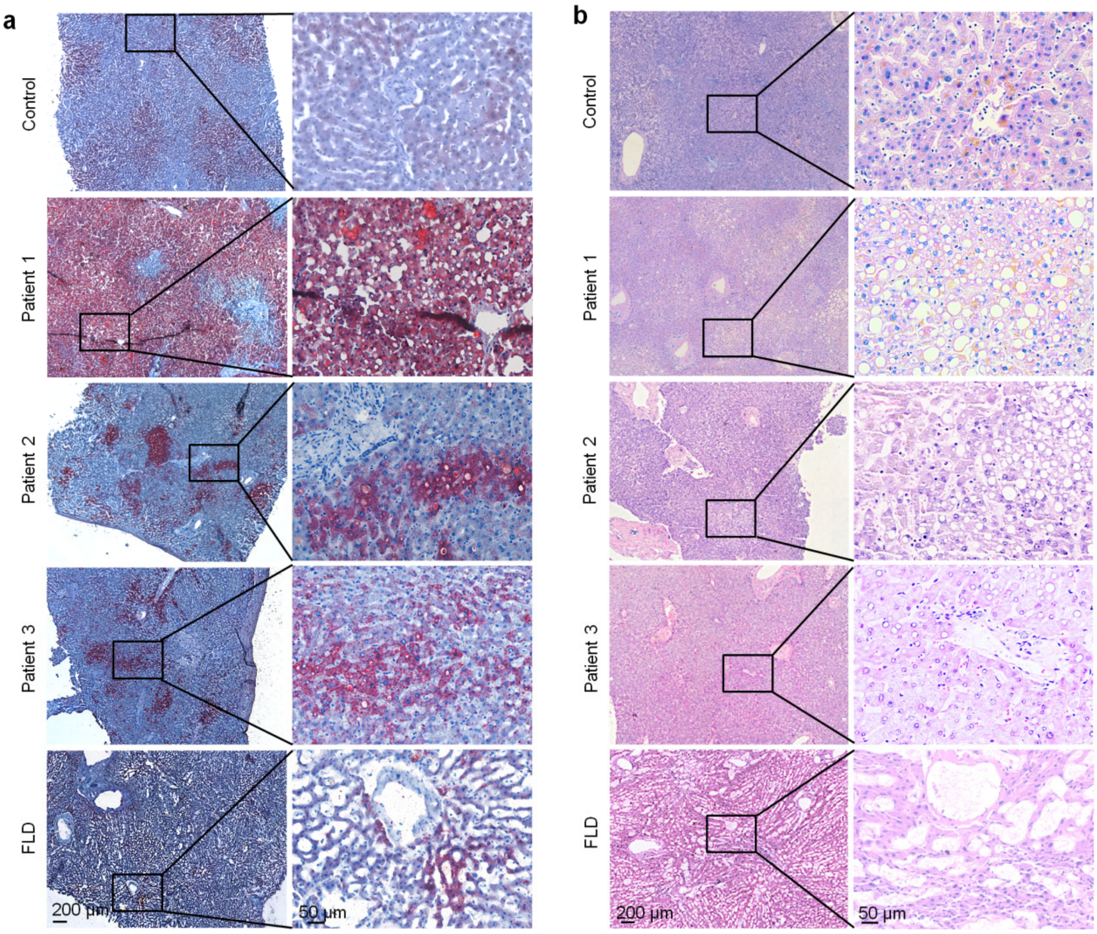

Fig. S7 Fat accumulation in the liver tissue of patients diagnosed with COVID-19. Oil red O staining (**a**) and H&E staining (**b**) of three samples of liver tissues of patients diagnosed with COVID-19, FLD, and control individuals (scale bar: 50 and 200 μm).

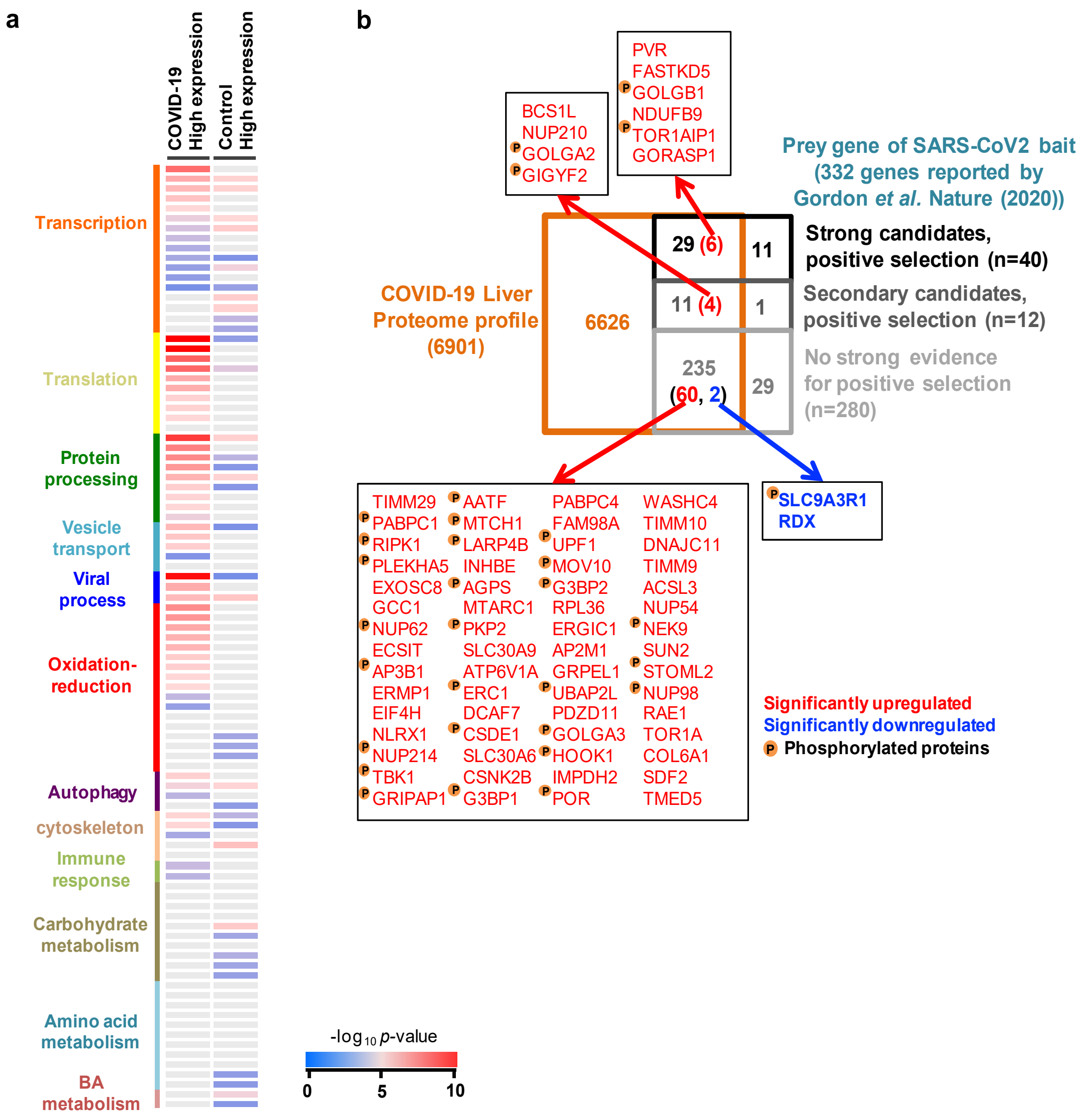

Fig. S8 Biological process analysis of the liver tissue of patients diagnosed with COVID-19. **a** Heatmap of significantly differential expression in the liver tissue of the COVID-19 and control groups. Columns with different colors on the left of the heatmap represent different function categories. Red and blue boxes indicate the -log$_{10}$ *p*-value of the enrichment analysis of enriched or depleted proteins, respectively. **b** Venn diagrams showing overlap of proteins in the liver of patients diagnosed with COVID-19 and reporting the interacting proteins of SARS-CoV-2. Red and blue fonts represent the number of significantly upregulated and downregulated proteins in liver tissues of patients diagnosed with COVID-19, respectively.

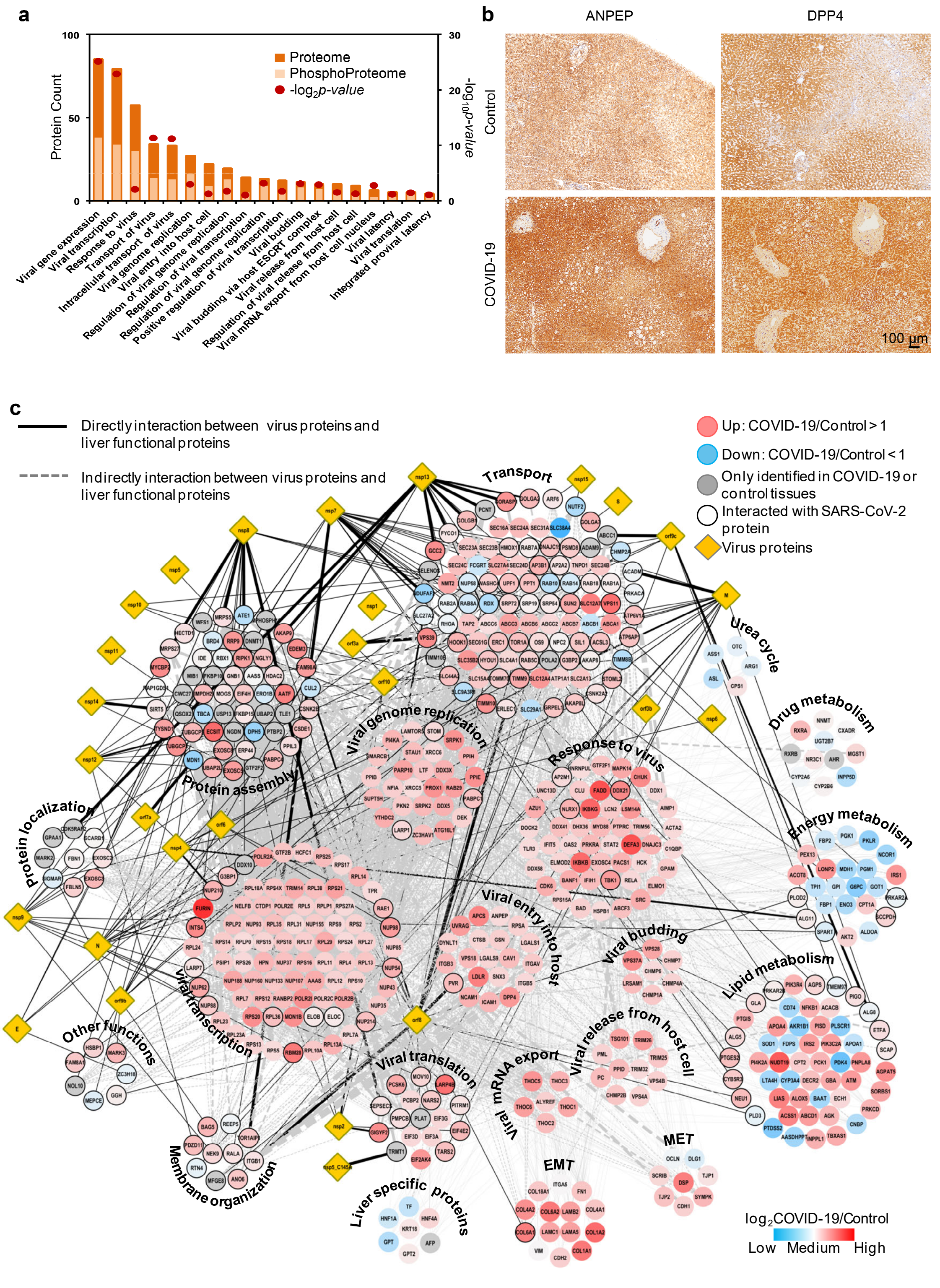

Fig. S9 Interaction network revealing the viral process characterization in the liver tissue of patients with COVID-19. **a** Biological insight into the virus process according to protein count and -log$_2$ *p*-value of the enrichment analysis. **b** Immunohistochemistry of ANPEP and DPP4 in liver tissues from patients with COVID-19 and control individuals

(scale bar: 100 μm). **c** Interaction network of proteins that are differentially expressed in the livers of patients diagnosed with COVID-19 vs. control liver tissue and SARS-CoV-2 proteins. Primary biological process analyses include a map of the functional categories. Red, blue, and gray circles represent proteins with high, low, and no expression in the liver of patients with COVID-19 compared to control livers, respectively; color-depth indicates the high and low level of protein abundance ratio ($\log_2$ COVID-19/Control). Yellow diamond represents the structural, non-structural, and additional open reading frame proteins of SARS-CoV-2. The circles with black edges represent the reported virus-interacting proteins. Black solid lines represent the interactions between the virus proteins and the reported virus-interacting proteins. Grey dotted lines represent the interactions between the reported virus-interacting proteins and other proteins.

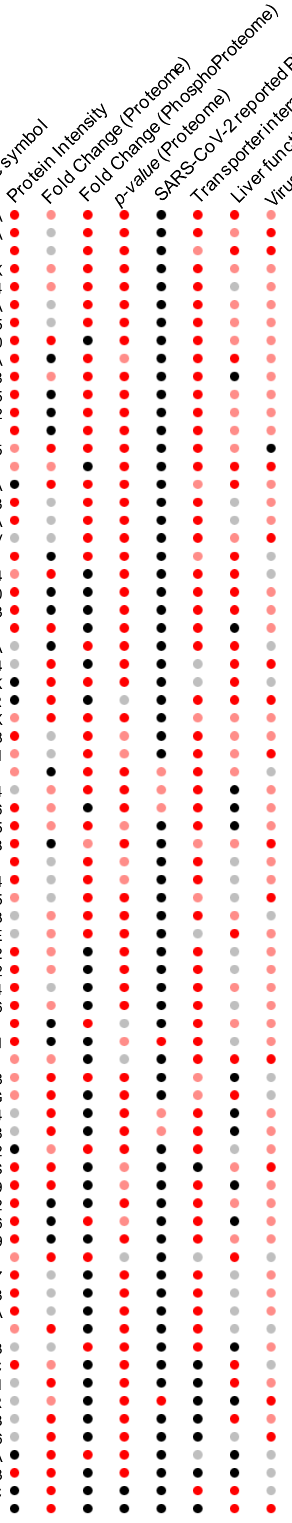

Rank	Gene symbol	A	B	C	D
1	RPL13A	5	2	0	1
2	RPSA	5	1	1	1
3	LGALS1	5	1	1	1
4	RPS4X	4	3	0	1
5	RPL14	4	2	1	1
6	RPL10A	4	2	1	1
7	RPL5	4	2	1	1
8	RPS20	4	2	0	2
9	RPS27A	4	2	0	2
10	RPL38	4	2	0	2
11	RPS5	4	2	0	2
12	RPS2	4	2	0	2
13	RPL11	4	2	0	2
14	DDX5	4	2	0	2
15	ICAM1	4	2	0	2
16	POLR2A	4	2	0	2
17	RPL23	4	1	2	1
18	RPL18A	4	1	2	1
19	ITGAV	4	1	2	1
20	HSPB1	4	1	1	2
21	MAPK14	4	1	1	2
22	RPLP0	4	1	0	3
23	RPS13	4	1	0	3
24	RPS21	4	1	0	3
25	RELA	4	0	2	2
26	DPP4	4	0	2	2
27	CHUK	4	0	2	2
28	LDLR	4	0	1	3
29	DDX3X	3	4	0	1
30	RPS18	3	3	1	1
31	GSN	3	3	1	1
32	AP2M1	3	3	1	1
33	NUP214	3	3	1	1
34	RPL36	3	3	0	2
35	RPS25	3	3	0	2
36	CTSB	3	3	0	2
37	RPLP1	3	2	2	1
38	RPS14	3	2	2	1
39	TRIM25	3	2	2	1
40	ABCF3	3	2	2	1
41	POLR2E	3	2	2	1
42	RPLP2	3	2	1	2
43	RPS12	3	2	1	2
44	RPL4	3	2	1	2
45	RPS16	3	2	1	2
46	RPL31	3	2	1	2
47	STOM	3	2	1	2
48	CAV1	3	2	1	2
49	VPS28	3	2	1	2
50	IKBKG	3	2	1	2
51	NUP54	3	2	1	2
52	NUP98	3	2	1	2
53	RANBP2	3	2	1	2
54	APCS	3	2	0	3
55	RPL29	3	2	0	3
56	RPL12	3	2	0	3
57	RPS26	3	2	0	3
58	RPS9	3	2	0	3
59	AZU1	3	1	3	1
60	RPL7	3	1	2	2
61	RPL13	3	1	2	2
62	RPS15A	3	1	2	2
63	TSG101	3	1	2	2
64	NUP93	3	1	2	2
65	PC	3	1	1	3
66	POLR2I	3	1	1	3
67	PVR	3	1	1	3
68	POLR2B	3	1	1	3
69	TRIM26	3	0	2	3
70	LSM14A	3	0	2	3
71	DEFA3	3	0	1	4
72	SRC	3	0	1	4
73	NCAM1	3	0	0	5

Terms	Description	A ● (red)	B ● (pink)	C ● (grey)	D ● (black)	Note
**Protein Intensity**	Average Intensity of protein expressed in COVID-19 liver sample	Rank: 1~51	Rank: 52~101	Rank: 102~152	Rank: 1 53~202	All involved proteins are arranged according to the average intensity of expression intensity (from high to low)
**Fold Change (Proteome)**	$\log_2$COVID-19/Control of protein	Rank: 1~51	Rank: 52~101	Rank: 102~152	Rank: 153~202	All involved proteins are arranged according to the ratio of COVID/Control (from high to low)
**Fold Change (PhosphoProteome)**	$\log_2$COVID-19/Control of phosphorylation site	FC>2, or the phosphorylation sites are unique identified in COVID-19 samples	2≥FC>1	1≥FC>0	The phosphorylation sites are unique identified in normal samples, or no phosphorylation site is identified in all samples.	--
***p-value* (Proteome)**	Benjamini-Hochberg adjusted *p-value* of protein in proteome profile dataset	p<0.01	0.05>p>0.01	0.10>p>0.05	p>0.1	--
**SARS-CoV-2 reported PPIs**	The protein that reported has interactions with SARS-CoV-2 proteins by Gordon et al. (Nature 2020).	Strong candidates and Secondary candidates	No strong evidence	--	No reported interactions with SARS-CoV-2 proteins	--
**Transporter interaction**	The number of interacted transporters	>3	2~3	1	0	The transporters refer to proteins involved in the transport module in Fig. S9c.
**Liver function interaction**	The number of interacted liver function related proteins	>3	2~3	1	0	The liver function related proteins including the proteins involved in lipid metabolism, energy metabolism, drug metabolism, urea cycle and liver specific proteins modules in Fig. S9c.
**Virus related biological process**	Virus related biological process that the proteins are involved.	Viral entry into host cell	Viral gene expression; viral genome; replication; viral transcription; viral translation	Viral latency; establishment of integrated proviral latency; viral budding; viral budding via host ESCRT complex; viral release from host cell; transport of virus; intracellular transport of virus; response to virus.	Positive regulation of viral transcription; positive regulation of viral genome replication; regulation of viral genome replication; regulation of viral transcription; viral mRNA export from host cell nucleus; regulation of viral release from host cell.	Virus related biological processes are shown in Fig. S9a.

Fig. S10 A scoring system for potential therapeutic targets in the liver for treating COVID-19. A scoring system table (top 73) of potential drug targets for liver injury treatment of patients with COVID-19 according to eight items. Circles of different colors represent different grading levels (A, B, C, and D) of each item. Red and blue

boxes indicate the numbers of each level score (circles of different colors) enriched in the COVID-19 group. The description of each item and the specific meaning of the level are on the right side of the scoring table.

Supplementary tables

Supplementary Table S1. Overview of the characteristics of patients diagnosed with COVID-19 involved in the study.

**Patient NO.**	**1**	**2**	**3**
**Basic information**			
**Gender**	Female	Female	Male
**Age**	66	55	83
**Virus nucleic acid testing**	Positive	Positive	Positive
**Comorbidities:**<br>**1. Diabetes**<br>**2. Hypertension**<br>**3. Liver disease: Liver comorbidities include non-alcoholic fatty liver disease, alcoholic liver disease and chronic hepatitis B**	2	None	2
**Initial symptoms: Fever/Cough**	Fever (38°C), fatigue, stuffy nose, stuffy chest, dyspnea, oxygen saturation (54%)	Fever (39.7°C), fatigue, stuffy chest, cough, chest pain, chilly oxygen saturation (56%)	Fever (36.8°C), sore throat
**Chest initial diagnosis**	Chest CT shows bilateral lung inflammation. The breath sounds of both lungs are thick with moist rales.	Chest CT shows bilateral pulmonary patchy lesions. The breath sounds of both lungs are thick, without obvious dry and wet rales.	Chest CT shows bilateral lung a few patchy lesions. The breath sounds of both lungs are clear, without obvious dry and wet rales.
**Drug-use: Antibiotics, NSAIDs, Ribavirin, Oseltamivir, Herbal medications, Interferon, Lopinavir/ritonavir or others**	Vancomycin, meropenem, nitroglycerin, immunoglobulin, albumin, methylprednisolone, adrenaline	Ceftriaxone, ribavirin, methylprednisolone, interferon, Coriolis, moxifloxacin, c-ball	Immunoglobulin, abidol, hormone
**Tissue location**	Upper left lateral lobe	Upper left lateral lobe	Upper left lateral lobe
**Blood biochemical indexes**			

Patient NO.	1	2	3
ALT (U/L)* **Reference interval: 7.00-40.00**	175.00	33.00	14.60
**AST (U/L)** **Reference interval: 13.00--35.00**	273.00	83.00	19.20
**ALB (g/L)** **Reference interval: 40.00-55.00**	29.80	34.60	30.60
**ALP (U/L)** **Reference interval: 50.00-135.00**	98.00	91.00	68.00
**GLU (mmol/L)** **Reference interval: 3.90-6.10**	7.70	18.60	6.01
**HDLC (mmol/L)** **Reference interval: 1.29-1.55**	0.34	0.34	1.39
**LDLC (mmol/L)** **Reference interval: 2.10-3.37**	1.46	1.04	2.84
**APOA (g/L)** **Reference interval: 1.00-1.60**	0.66	0.50	1.01
**APOB (g/L)** **Reference interval: 0.60-1.10**	1.29	0.90	1.07
**TG (mmol/L)** **Reference interval: 0.51-1.70**	7.69	6.62	0.88

Supplementary Table S2-1. All Proteins identified in COVID-19 and control liver samples.

Supplementary Table S2-2. All phosphorylation sites identified in COVID-19 and control liver samples.

Supplementary Table S3-1. Differentially expressed proteins between COVID-19 and control liver samples.

Supplementary Table S3-2. Differentially expressed phosphorylation sites between COVID-19 and control liver samples.

Supplementary Table S4-1. Protein-protein interactions between SARS-CoV2 proteins and the proteins identified in the COVID-19 samples in Fig. S9c.

Supplementary Table S4-2. Detailed protein-protein interactions about the proteins involved in Fig. S9c.

Supplementary Table S4-3. Detailed information about the proteins involved in the protein-protein interaction network (Fig. S9c).

Supplementary Table S5. Detailed scoring table for potential therapeutic targets in liver treatment of the patients diagnosed with COVID-19.

Supplementary Table S6. Companies providing equipment, reagents and/or supplies.
